# Correlation between penile cuff test and pressure-flow study in patients candidates for trans-urethral resection of prostate

**DOI:** 10.1186/1471-2490-14-103

**Published:** 2014-12-19

**Authors:** Daniele Bianchi, Angelo Di Santo, Gabriele Gaziev, Roberto Miano, Stefania Musco, Giuseppe Vespasiani, Enrico Finazzi Agrò

**Affiliations:** School of Specialization in Urology, University of Rome Tor Vergata, Viale Oxford, 81-00133 Rome, Italy; NeuroUrology Unit, IRCCS Fondazione Santa Lucia, Rome, Italy; Department of Experimental Medicine and Surgery, University of Rome Tor Vergata, Rome, Italy; Neuro-Urology Unit, Careggi Hospital, Florence, Italy

**Keywords:** Bladder isovolumetric pressure, Non-invasive urodynamics, Penile cuff test, Prostate, Trans-urethral resection of prostate

## Abstract

**Background:**

Aim of this study was to make a comparison between penile cuff test (PCT) and standard pressure-flow study (PFS) in the preoperative evaluation of patients candidates for trans-urethral resection of prostate (TURP) for benign prostatic obstruction (BPO).

**Methods:**

We enrolled male patients with lower urinary tract symptoms candidates for TURP. Each of them underwent a PCT and a subsequent PFS. A statistical analysis was performed: sensitivity (SE), specificity (SP), positive predictive value (PPV), negative predictive value (NPV), likelihood ratio and ratio of corrected classified were calculated. Fisher exact test was used to evaluate relationships between PCT and maximal urine flow (*Q*_max_): a *p-*value < 0.05 was considered statistically significant.

**Results:**

We enrolled 48 consecutive patients. Overall, at PCT 31 patients were diagnosed as obstructed and 17 patients as unobstructed. At the subsequent PFS, 21 out of 31 patients diagnosed as obstructed at PCT were confirmed to be obstructed; one was diagnosed as unobstructed; the remaining 9 patients appeared as equivocal. Concerning the 17 patients unobstructed at PCT, all of them were confirmed not to be obstructed at PFS, with 10 equivocal and 7 unobstructed. The rate of correctly classified patients at PCT was 79% (95%-CI 65%-90%). About detecting obstructed patients, PCT showed a SE of 100% and a SP of 63%. The PPV was 68%, while the NPV was 100%.

**Conclusions:**

PCT can be an efficient tool in evaluating patients candidates for TURP. In particular, it showed good reliability in ruling out BPO because of its high NPV, with a high rate of correctly classified patients overall. Further studies on a huger number of patients are needed, including post-operative follow-up as well.

## Background

The role of urodynamics (UD) in the diagnosis of benign prostate obstruction (BPO) has been intensively investigated [1, 2].

In clinical practice, when required, a proper evaluation and quantification of BPO is performed by invasive UD, in particular pressure-flow study (PFS) [1].

Over the last two decades, some alternative, less invasive tests have been proposed [3], based on equipment consisting of an external condom catheter [4], an intra-urethral device [5] or an inflatable cuff around the penis – penile cuff test (PCT) – with inflation-deflation cycles [6].

Instead of the direct intravesical sampling used in PFS, non-invasive UD aims to give information about bladder pressure by evaluating the equal urine pressure either along the urethra (in penile cuff), or at the external meatus (in external condom catheter).

In the PCT with inflation-deflation cycles [6], the pressure needed to stop the flow (*p*_cuff_) represents the bladder isovolumetric pressure (BIP) e.g. the bladder pressure during an isovolumetric contraction. This pressure is detected by a cuff placed around the penis before micturition [6].

**Figure 1:**
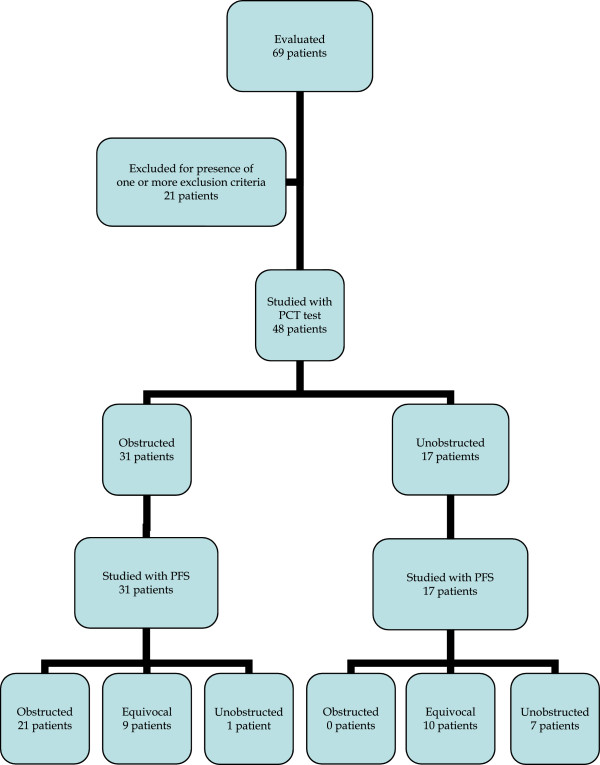
**The principal of the test is similar to bladder pressure measurement.** A small cuff is placed around the penis. When micturition has commenced, the cuff is inflated. The cuff pressure required to stop flow should equal bladder pressure. p iso: Isovolumetric pressure.

The cuff is automatically inflated during the voiding phase, in order to stop urine flow, and then deflated again. The inflation-deflation cycle is repeated several times during a single micturition, thus allowing to correctly assess BIP (see Figure 1).

PCT results can be plotted on the nomogram proposed by Griffiths [7] which is designed on a cartesian plane with maximal urine flow (*Q*_max_) on the *x*-axis and *p*_cuff_ on *y*-axis, with an ascending straight line, with *y*-intercept equal to 80 cm *H*_*2*_*O*, separating obstructed from non-obstructed patients.

Recently, a new prototype of PCT has been proposed [8], using an automatically controlled inflatable cuff which detects bladder voiding pressure at constant low urine flow instead of inflation-deflation cycles.

The purpose of this study was to compare the data of PCT with inflation-deflation cycles with those of a standard PFS in patients candidates for trans-urethral resection of prostate (TURP).

## Methods

Male patients who previously received indication to undergo a TURP in our or in a different center were included. Indication for TURP had been made on referral urologist opinion, generally on the basis of the presence of lower urinary tract symptoms (LUTS) and a reduced flow rate, independently by other aspects as prostate volume and alpha-blockers effectiveness.

A urine sample for urine culture was collected by spontaneous micturition within 7 days before the tests in order to rule out possible infections. Exclusion criteria were diabetes mellitus, any neurological disease, use of drugs impairing bladder contractility or impacting on lower urinary tract function, an indwelling bladder catheter over the previous six months, presence of urinary tract infection, suspect of malignancies.

Approval of the study by Ethics Committee of Policlinico Tor Vergata was obtained, and all patients signed a written informed consent to be included. For each patient, we performed a PCT followed by a subsequent PFS and both procedures were conducted by the same urodynamicist.

PCT was performed by Mediplus CT3000 Cuff Machine®, which allows multiple inflation-deflation cycles during a single micturition, getting several BIP measurements.

The patients were instructed to perform a micturition without abdominal straining.

For each inflation cycle we applied the exclusion rules proposed by Drinnan et al. [9], thus a cycle was immediately excluded in case of one of the following conditions:

-  No flow recovery after cuff deflation, meaning that the micturition ended during the last cycle, so the cuff pressure could have been not responsible for urine flow stop;
-  There was an ‘erratic’ flow trace, which could be related to straining or maybe to contractions by the pelvic floor or membranous urethra;
-  The urine flow was not interrupted at the device maximum pressure, which is set at 200 cm *H*_*2*_*O* for safety reasons. This situation is associated with highly contractile bladder.

Furthermore, we repeated any test showing a total bladder volume less than 150 mL.

PFS was performed by a urodynamic equipment (Life-Tech®, Stafford, TX, USA) with water-filled bladder catheter and rectal balloon (Life-Tech®, Stafford, TX, USA), after a filling phase with non-physiological filling rate (30–50 mL/s).

The examination was conducted according to the *International Continence Society* recommendations [10, 11].

PFS data were plotted on the Abrams-Griffiths modified nomogram [12, 13], while PCT results were plotted on the nomogram proposed by Griffiths [7].

For each of two categories – obstructed versus non-obstructed – patients were subdivided into two subgroups according to their *Q*_max_, with a threshold of 10 mL/s [14], in order to evaluate if *Q*_max_ was able to improve accuracy.

Sensitivity (SE), specificity (SP), positive predictive value (PPV), negative predictive value (NPV), likelihood ratio and ratio of corrected classified were calculated.

In order to assess accuracy of estimated value, 95% confidence interval (95%-CI) was calculated for SE, SP, PPV, NPV and ratio of corrected classified.

Fisher exact test was used to evaluate relationships between PCT and *Q*_max_: a *p-*value < 0.05 was considered statistically significant.

Software Stata 13.0 (College Station®, Texas) was used for all analysis.

Results have been reported according to *Standards for Reporting of Diagnostic accuracy* (STARD) flow-chart [15].

The research was carried out in compliance with the Helsinki Declaration - Ethical Principles for Medical Research Involving Human Subjects.

## Results

We enrolled 48 consecutive male patients – mean age 61.5 ± 13.1 years.

On uroflowmetry, median *Q*_max_ was 11.6 mL/s (range 4.0-25.0 mL/s), with a median post-void residual urine volume (PVR) of 42 mL (range 0–430 mL) detected by ultrasound scan. PSA mean value was 2.35 ng/mL (range 0.80-4.10 ng/mL).

No adverse events occurred during or after the tests.

Overall, at PCT 31 patients were diagnosed as obstructed and 17 patients as unobstructed on Griffiths nomogram [7].

On the subsequent PFS, according to Abrams-Griffiths nomogram [12, 13], 21 out of 31 patients diagnosed as obstructed at PCT were confirmed to be obstructed; one was diagnosed as unobstructed; the remaining 9 patients appeared as equivocal.

**Figure 2:**
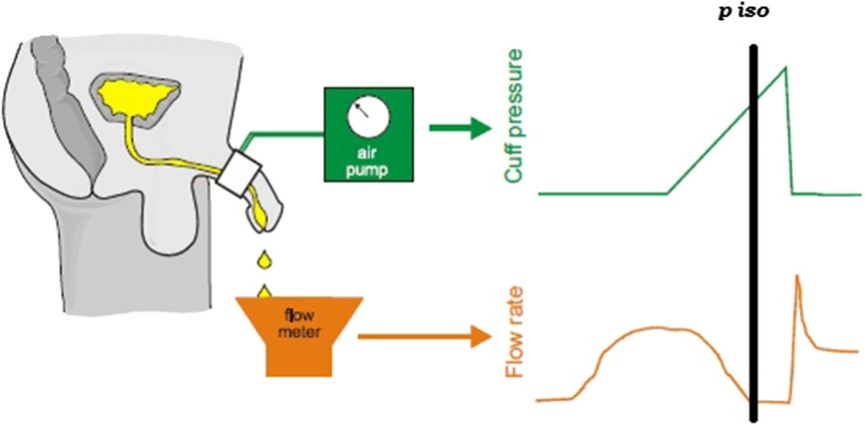
**Results according to** ***Standards for Reporting of Diagnostic accuracy*** **(STARD) flow-chart.** PCT: Penile Cuff Test. PFS: Pressure-Flow Studies.

Concerning the 17 patients unobstructed at PCT, all of them were confirmed not to be obstructed on PFS, with 10 equivocal and 7 unobstructed (see Table 1 and Figure 2 for STARD flow-chart).

**Table 1:** **Results of penile cuff test (PCT) compared with pressure-flow studies (PFS)**

	PFS obstructed	PFS Unobstructed/Equivocal	Total
PENILE CUFF obstructed	21	10	31
PENILE CUFF unobstructed	0	17	17
Total	21	27	48

The rate of correctly classified patients at PCT was 79% (95%-CI 65%-90%).

About pressure measurements, overall at PCT we obtained a mean *p*_cuff_ equal to 133.75 cm *H*_*2*_*O* (SD 33.45 cm *H*_*2*_*O*), while at PFS we had a mean detrusorial pressure at *Q*_max_ flow equal to 52.69 cm *H*_*2*_*O* (SD 21.94 cm *H*_*2*_*O*).

Focusing on obstructed patients, at PCT we had a mean *p*_cuff_ =157.00 cm *H*_*2*_*O* (SD 26.83 cm *H*_*2*_*O*), while at PFS the mean detrusorial pressure was 74.00 cm *H*_*2*_*O* (SD 14.13 cm *H*_*2*_*O*).

With regard to the further subdivision according to *Q*_max_, 15 patients out of the 31 obstructed at PCT showed a *Q*_max_ < 10 mL/s, with the other 16 patients having a *Q*_max_ ≥ 10 mL/s.

On the other hand, among the 17 unobstructed patients on PCT, we had 5 with a *Q*_max_ < 10 mL/s and 12 with a *Q*_max_ ≥ 10 mL/s.

Patients categorization into subgroups according to their *Q*_max_ greater or less than 10 mL/s did not produce a further improvement of PCT ability to diagnose BPO (Fisher exact test, *p* = 0.2362).

About detecting obstructed patients, PCT showed a SE of 100% (95%-CI 84-100%) and a SP of 63% (95%-CI 42-81%), with a positive likelihood ratio of 2.7 (95%-CI 1.65-4.42). The PPV was 68% (95%-CI 49-83%), while the NPV was 100% (95%-CI 80-100%). Results are summarized in Table 2.

**Table 2:** **Results of penile cuff test (PCT) compared with pressure-flow studies (PFS)**

PCT O		PCT U						
PFS O (n)	PFS U (n)	PFS O (n)	PFS U (n)	SE %	SP %	PPV %	NPV %	LR + %
21	10	0	17	100,0	63,0	67,7	100,0	2,7

PCT: Penile Cuff Test.

PFS: Pressure-Flow Studies.

O: Obstructed.

U: Unobstructed.

n: Number of patients.

SE: Sensitivity.

SP: Specificity.

PPV: Positive Predictive Value.

NPV: Negative predictive Value.

LR+: Positive Likelihood Ratio.

## Discussion

Over the last century, a simple evaluation of PVR has been proposed as an appealing tool for diagnosing BPO. Nevertheless, a huge PVR may be due to an impaired detrusor contractility (IDC) [16]. In fact, it has been pointed out that up to one half of unobstructed patients with LUTS could have elevated PVR, while up to one forth of severely obstructed patients could show no PVR [1].

Thus, the association between elevated PVR and BPO is not strong enough to be used as a useful clinical tool [17].

In some papers, the role of uroflow trace has been investigated as well, but no reliable relation between its profile and BPO was found out [18].

Some Authors have shown that uroflowmetry could be able to assess the presence of BPO in the vaste majority of patients with *Q*_max_ less than 10 mL/s, with a progressively decreasing rate of BPO in case of Q_max_ major than 10 mL/s [1, 2, 14].

Accordingly, *European Association of Urology* guidelines have considered PFS as an optional test before surgery for BPO, usually indicated in the preoperative evaluation of patients showing a *Q*_max_ > 15 mL/s [19].

Conversely, 25-30% of men with decreased *Q*_max_ at uroflowmetry are unobstructed [1]. Indeed, decreased uroflow can result from either impaired detrusor contractility (IDC) or BPO; thus, only detrusor pressure measurement is able to distinguish between those conditions [18, 20].

Furthermore, there are no tips on uroflowmetry trace shape that allows a distinction between BPO and IDC [18]; on the other hand, a normal uroflow test does not rule out BPO [20].

As a consequence, PFS still represents the gold standard for a proper evaluation of BPO in male patients, above all when an IDC is suspected [1].

In clinical practice, the nomogram proposed by Abrams and Griffiths for the diagnosis of obstruction in males at PFS has been widely used [16].

A further nomogram proposed by Abrams [13] aims to give a more accurate patients categorization by the introduction of bladder outlet obstruction index (BOOI) and bladder contractility index (BCI).

Thus, PFS represents the gold standard for the evaluation of BPO [1]. Nevertheless, this test is not used as a routine examination before surgery for BPO [19], because it is considered time-consuming, not cost-effective overall [1] and a potential cause of morbidity [21].

Over the last 30 years, no simple tool proved to be reliable in distinguishing between BPO and IDC.

The role of non-invasive UD in clinical practice is still unclear [19] and few data have been published about correlation between PCT and PFS findings [6, 7, 22, 23].

Aim of our study was to make a comparison between PCT and PFS in the diagnostic work-up on patients candidates for TURP: summarizing our results, PCT showed a SE of 100% and a SP of 63% in detecting obstructed patients, with a PPV of 68% and a NPV of 100%.

Overall, the rate of correctly classified patients at PCT was 79%. In particular, non-obstructed patients at PCT were confirmed as non-obstructed at PFS.

Using the nomogram modified for non-invasive pressure measurement, Griffiths et al. [7] obtained with PCT a PPV of 68% and a NPV of 78% for PFS diagnosis of BPO. Besides, they noticed that predictive accuracy for obstruction could be improved by the additional criterion of *Q*_max_ less than 10 mL/s, thus obtaining a PPV of 88% and a NPV of 86%.

In our study, patients categorization into subgroups according to their *Q*_max_ (threshold 10 mL/s) did not add any further information as it did not get confirmation of its statistical significance at Fisher exact test.

The difference between Griffiths’ and our results could be due to a different selection of patients: in our study, only patients who were candidates for a TURP [24] were enrolled, while in Griffiths’ paper the Authors intended to analyze patients complaining with LUTS [7]. Nevertheless, further studies are needed to investigate this aspect.

Our data seem to confirm those ones obtained in another, more recent paper based on 30 consecutive patients complaining with LUTS. In this study, Borrini L et al. [22] found for PCT a PPV of 82% and a NPV of 88% for BPO at PFS.

According to our experience, non-invasive UD, in particular PCT, can be a useful diagnostic tool in patients candidates for TURP, suggesting a possible solution to the thorny problem about urodynamic tests before surgery for BPO. In fact, compared to PFS, PCT appeared as a quick and accurate test to rule out a BPO condition because of its high NPV. Thus it could be used to run a selection of non-obstructed patients suspected for an eventual IDC condition. Indeed, such patients are the most critical ones in BPO surgery [25], with some papers reporting about one forth of them showing no symptoms improvement after a surgical treatment [24, 26].

In a paper by Harding et al. [27], a consecutive cohort of 208 men undergoing TURP were previously evaluated by PCT: 87% of patients diagnosed with BPO had a clinical improvement after surgery, while only 56% of patients deemed as not obstructed had a good outcome.

By such diagnostic pathway, non-obstructed patients (probably with IDC) could be easily recognized and adequately counseled in advance about the prospect of poor or partial symptoms improvement after surgery for BPO, avoiding a PFS.

Furthermore, the rate of correctly classified patients at PCT was high, confirming that most obstructed patients can be adequately evaluated by PCT.

We should also consider that PCT categorization does not allow for ‘equivocal’ patients, who finally represent the mismatch between the two urodynamic tests. Anyway, those patients can be mostly considered eligible to surgery, so PFS could be neglected in such cases.

Only patients with an unclear diagnosis could be suggested to undergo PFS, while the other ones could be probably evaluated just by PCT, getting the amount of pre-operative information useful both to the surgeon and to the patient, in terms of preoperative counseling.

Limitations of our study are the relatively small sample size and the lack of a post-operative follow-up to assess TURP efficacy in different categories of patients.

On the other hand, this is, to our knowledge, the first study comparing PCT to PFS in patients candidates for TURP.

Further papers on large series of patients including post-operative follow-up are needed, in order to assess the real role of PCT in the pre-operative evaluation for BPO.

## Conclusions

PCT can be an efficient tool in evaluating patients candidates for TURP. In particular, it showed good reliability in ruling out BPO because of its high NPV, with a high rate of correctly classified patients overall. Further studies based on a bigger sample size are needed, including post-operative follow-up.
